# Hydrogen Sulfide: A Versatile Molecule and Therapeutic Target in Health and Diseases

**DOI:** 10.3390/biom14091145

**Published:** 2024-09-10

**Authors:** Aqsa Shahid, Madhav Bhatia

**Affiliations:** Department of Pathology and Biomedical Science, University of Otago, Christchurch 8140, New Zealand; shaaq042@student.otago.ac.nz

**Keywords:** hydrogen sulfide, H_2_S donors, H_2_S inhibitors, inflammatory conditions, cardiovascular diseases, viral infections, neurological disorders, therapeutic applications, molecular pathways

## Abstract

In recent years, research has unveiled the significant role of hydrogen sulfide (H_2_S) in many physiological and pathological processes. The role of endogenous H_2_S, H_2_S donors, and inhibitors has been the subject of studies that have aimed to investigate this intriguing molecule. The mechanisms by which H_2_S contributes to different diseases, including inflammatory conditions, cardiovascular disease, viral infections, and neurological disorders, are complex. Despite noteworthy progress, several questions remain unanswered. H_2_S donors and inhibitors have shown significant therapeutic potential for various diseases. This review summarizes our current understanding of H_2_S-based therapeutics in inflammatory conditions, cardiovascular diseases, viral infections, and neurological disorders.

## 1. Introduction

This review aims to understand the role of H_2_S distribution, biosynthesis, mechanistic pathways, and H_2_S-based therapeutics in different diseases—inflammatory conditions, cardiovascular disorders, viral diseases, and central nervous system diseases. In modern biology, a fascinating theme is that small molecules can contribute to intricate physiological and pathological functions in living organisms [1]. Hydrogen sulfide (H_2_S) was traditionally believed to be a toxic, flammable gas with an unpleasant rotten egg odor [2]. However, recent research has unveiled that H_2_S is an important cellular signaling molecule, marking a significant paradigm shift in research. The recognition of H_2_S as an important contributor to both physiological and pathological processes underscore the importance and impact of this gas. H_2_S is the third gasotransmitter, followed by nitric oxide (NO) and carbon monoxide (CO). It exhibits various physiological actions, including apoptosis, cytoprotection, vasodilation, angiogenesis, modulation of inflammatory responses, antioxidant properties, regulation of vascular tone, enhancing cell survival, and regulation of cellular metabolism [3,4,5]. Pathological conditions, including inflammatory conditions (such as arthritis/joint inflammation, sepsis, and acute pancreatitis), cardiovascular disorders, viral diseases, and central nervous system diseases, may arise due to the disruption in its endogenous production or metabolism. By examining the underlying mechanisms and therapeutic potential of H_2_S donors and inhibitors, this article summarizes our current understanding of the role of H_2_S in health and disease.

## 2. Synthesis and Distribution of H_2_S

**Figure 1:**
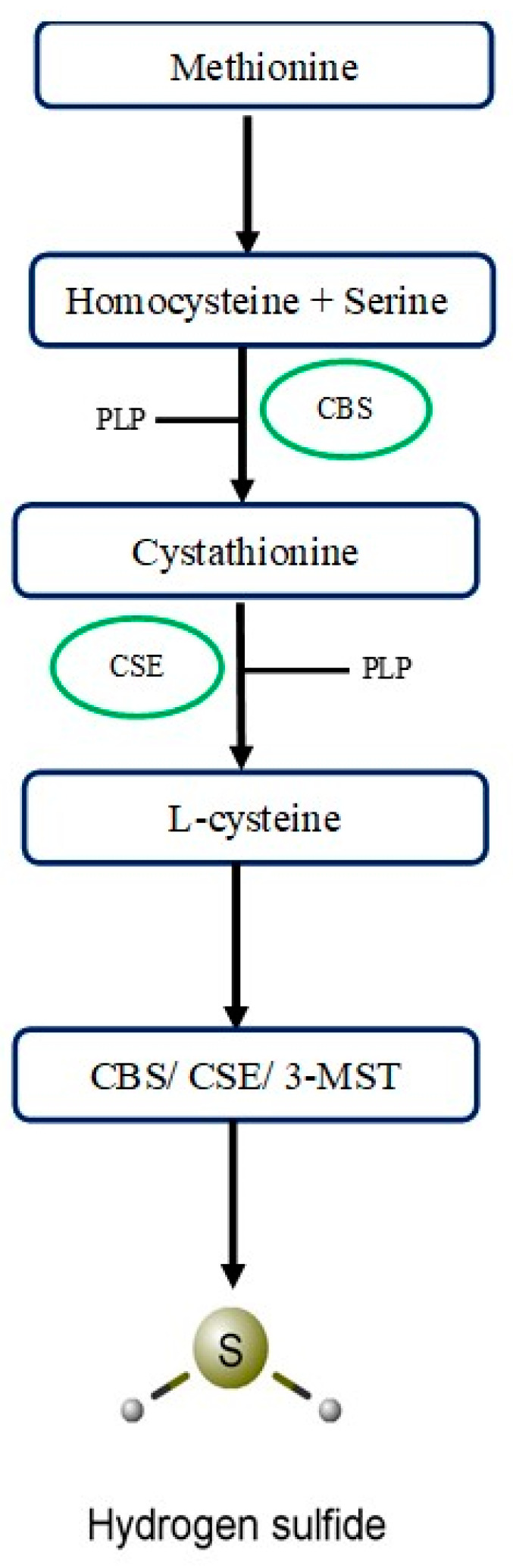
An overview of H_2_S biosynthesis. This pathway shows the biosynthesis of H_2_S. Pyridoxal 5’-phosphate (PLP) acts as a co-factor for Cystathionine β-synthase (CBS) and Cystathionine γ-lyase (CSE) dependent H_2_S synthesis.

Three enzymes—cystathionine-β-synthase (CBS), cystathionine-γ-lyase (CTH, CGL, or CSE), and 3-mercaptopyruvate sulfurtransferase (3-MST or MPST)—are primarily responsible for H_2_S biosynthesis (Figure 1). Some other enzymatic or non-enzymatic pathways can also produce H_2_S under normal or pathological conditions.

### 2.1. Cystathionine-β-Synthase

CBS is also termed as beta-thionase, serine sulfhydrase, and L-serine hydrolyase. This enzyme is encoded by the cystathionine-β-synthase gene present on chromosome 21 in humans [6]. It is predominantly present in different parts of the brain (including the cerebral cortex, cortical astrocytes, corpus callosum, neuronal cells, hippocampus, olfactory bulb, and cerebellum) and in the pancreas (acinar cells) [7]. CBS is predominantly present in the cytosol, but it has also been found in mitochondria. Levels of CBS in mitochondria are increased after hypoxia [8].

### 2.2. Cystathionine-γ-Lyase

CSE is also referred to as homoserine dehydratase, cysteine desulfhydrase, cystine desulfhydrase, and γ-cystathionase. This enzyme in humans is encoded by the CSE gene present on chromosome 1. This gene is highly expressed in several organs, including the liver, lungs, uterus, and kidneys. A small amount of CSE contributes to H_2_S synthesis in the brain as well [9,10].

### 2.3. 3-Mercaptopyruvate Sulfurtransferase

3-MST is also called human liver rhodanese 2, β-mercaptopyruvate sulfurtransferase, thiosulfate sulfurtransferase 2, and tRNA thiouridin modification protein. This enzyme is encoded by a 3-mercaptopyruvate sulfurtransferase gene present on chromosome 22 in humans [11]. It has been reported in several studies that 3-MST is predominantly present in mitochondria. Studies from neurons, heart, liver, endothelial, and epithelial cells have shown that an equal concentration of 3-MST is present in cytosol as well as mitochondria [12,13].

Figure 1 provides an overview of the synthesis of H_2_S in the body.

### 2.4. Other Sources of H_2_S

In addition to these enzymatic sources, H_2_S can also be produced by gut microbiota and through different natural dietary sources. Natural sources, including sulforaphane and erucin (which are abundantly present in green vegetables) and diallyl disulfide and diallyl trisulfide (DATS), are found in garlic [14,15]. Some bacteria, including *Desulfotomaculum*, *Desulfovibrio*, and *Desulfobacter*, are abundantly present in the colon and are known as sulfate-reducing bacteria [16,17]. Many bacterial strains, such as *Staphylococcus*, *Salmonella*, *Escherichia coli*, *Corynebacterium*, and *Enterobacter*, also have sulfite reductase. Furthermore, several anaerobic bacterial species, e.g., *Clostridia* and *Bacillus*, also play a role in the conversion of cysteine into H_2_S, ammonia, and pyruvate through cysteine desulfhydrases [18].

**Figure 2:**
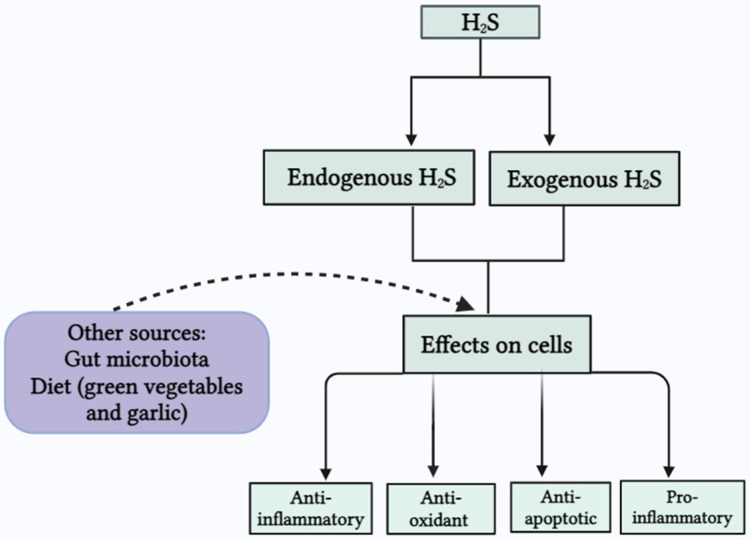
Endogenous and exogenous H_2_S and effects on cells.

Figure 2 provides an overview of the sources of H_2_S in the body and its effects on cells.

## 3. H_2_S Donors

H_2_S-releasing compounds (Table 1) are widely used to understand the biological pathways of H_2_S and develop H_2_S-targeting therapeutics. In recent decades, the development of H_2_S donors has been a rapidly expanding area of research [19,20]. These donors are designed to release H_2_S by various mechanisms. According to the literature, the role of H_2_S, using H_2_S donors, appears to be controversial. In some conditions, H_2_S has been shown to have pro-inflammatory actions, whereas some studies have pointed to the anti-inflammatory effects of H_2_S [21,22]. These differences in effects of H_2_S can be attributed to several factors such as the nature of H_2_S donors, experimental models, method of administration, timing, site, and dosage of the agent. H_2_S donors are classified into two classes—fast H_2_S-releasing compounds and slow-H_2_S-releasing compounds. Among fast H_2_S-releasing compounds inorganic sulfide salts, including sodium hydrogen sulfide (NaHS) and sodium sulfide (Na_2_S), are commonly used. These inorganic salts have protective effects in neurological and cardiovascular disorders but show pro-inflammatory and deleterious effects in conditions such as sepsis [23,24]. Despite their protective effect, the main problem with these H_2_S donors is their quick, complete, and uncontrollable release in an aqueous solution. This quick release results in the loss of H_2_S in solution, and therefore the efficacy of these salts is compromised [25,26]. Among slow-H_2_S-releasing compounds, perthiol-based donors, thioamino acids (thioglycine and thiovaline), photolabile H_2_S donors, thioamide- and aryl isothiocyanate-based donors, S-aroylthiooximes, S-propargyl-cysteine (SPRC), dithioperoxyanhydrides, morpholin-4-ium 4-methoxyphenyl-morpholino-phosphinodithioate (GYY4137, H_2_S-releasing NSAIDs, e.g., H_2_S-diclofenac (ACS-15), sodium thiosulfate (Na_2_S_2_O_3_), and garlic-derived sulfur-containing compounds diallyl disulfide (DADS), and allicin are used [27]. These compounds can be used for therapeutic purposes due to their reduced toxicity, effective biological functions, and controlled H_2_S release. H_2_S donors exert their effects through several mechanisms, such as combating oxidative stress, protein persulfidation, and interacting with metal-containing proteins. H_2_S has inherent antioxidative activities, which decrease the production of ROS by neutralizing oxidative stress. This activity preserves the redox balance between cells and prevents cell damage [28,29]. Persulfidation is a reversible biochemical process in which H_2_S interacts with cysteine in proteins to make persulfide bonds. This modification is similar to phosphorylation and nitrosylation, which regulate the structure and function of proteins [30]. Furthermore, H_2_S interacts with several metal-containing proteins, such as chromoprotein, ferritin, molybdenum protein, zinc protein, and cuproprotein. Interaction of H_2_S with these proteins can affect their conformation and function through different mechanisms. These mechanisms may include the modification of coordination number, the binding ability of metals, and oxidation state [31]. These mechanisms help in mitigating inflammatory, viral, cardiovascular, and neurological disorders. 

## 4. H_2_S Synthesis Inhibitors

To investigate the functions of H_2_S, scientists have used different approaches to inhibit H_2_S synthesis, i.e., by pharmacological agents or by H_2_S-producing gene silencing through siRNA. Different pharmacological agents, including aminooxyacetic acid (AOAA), hydroxylamine (HA), propargylglycine (PAG), trifluoroalanine, and β-cyanoalanine (BCA), are commonly used as H_2_S synthesis inhibitors [53,54]. Among these, AOAA is considered a specific inhibitor of CBS, whereas PAG and BCA selectively target the CSE activity. PAG exists in two isoforms, i.e., L-isomer and D-isomer. The L-isomer is used to inhibit CSE production, whereas the D-isomer is believed to be a nephrotoxin [55,56]. In addition to these conventional pharmacological inhibitors, novel H_2_S-targeting inhibitors, including aurintricarboxylic acid (NSC4056), S-3-carboxpropyl-L-cysteine (CPC), and 2-arylidene hydrazinecarbodithioates, have been developed. These compounds show significant inhibition of CSE, but not CBS, in a dose-dependent manner [57]. Furthermore, the expression of the CSE gene is inhibited through the small interfering RNA (siRNA). Our group has used siRNA as a targeted probe to suppress the expression of CSE in monocytes/macrophages, thereby reducing the production of H_2_S in a mouse model of sepsis. Using this approach, we showed that CSE gene silencing through siRNA inhibits H_2_S production in macrophages. Inhibition of H_2_S production by CSE gene silencing decreases the levels of pro-inflammatory chemokines and cytokines in the liver and lungs [58]. In addition to pharmacological inhibition and gene silencing, H_2_S synthesis can also be inhibited by some alternative pathways. These pathways include modulation in levels of cysteine (substrate for H_2_S synthesis) and co-factor S-adenosylmethionine (SAM), and decreasing the levels of homocysteine [59,60].

## 5. H_2_S in Inflammatory Conditions

Inflammation is a protective response of the body to harmful stimuli, including infection, irradiation, and poisonous chemicals, and functions by eliminating these harmful stimuli and starting the healing process [61,62]. Among fast H_2_S-releasing compounds, inorganic sulfide salts (NaHS and Na_2_S) show pro-inflammatory effects in sepsis by the upregulation of several pro-inflammatory mediators. On the other hand, slow H_2_S-releasing donors show anti-inflammatory effects by several intricate signaling pathways. These pathways include suppression of the release of inflammatory regulators, which mitigates the inflammatory response. Slow H_2_S-releasing donors act by inhibiting the activation of NF-κB. This inhibition decreases the release of inflammatory cytokines and reduces the infiltration of neutrophils to the site of the inflammation [63].

### 5.1. H_2_S and Arthritis/Joint Inflammation

Gout and rheumatoid arthritis (RA) are considered the most common forms of joint inflammation. The role of H_2_S in joint inflammation is controversial; in some cases, H_2_S acts as a pro-inflammatory mediator, while in others it has been reported to have anti-inflammatory effects. In clinical RA, some advancements have been made to understand the contribution of H_2_S, but more research is needed to clearly define its role. In order to address this gap in knowledge, our group has shown that H_2_S, has pro-inflammatory actions in clinical arthritis. Levels of H_2_S were elevated in the synovial fluid of patients with RA and gout when compared with plasma H_2_S levels in the same patients. Furthermore, in the case of RA, there was a significant correlation between synovial fluid H_2_S levels and the disease activity score. This find-ing underscores the critical role of H_2_S in clinical arthritis and points to its potential as a therapeutic target [64]. Another common form of arthritis that significantly affects elderly people in particular is osteoarthritis (OA) [65]. In hind-paw edema induced by carrageenan (a standard method to cause acute joint inflammation), increased levels of H_2_S synthesis with inflammation were observed. Prophylactic administration of PAG showed a decrease in hind-paw inflammation in a concentration-dependent manner [66]. Moreover, treatment with D-penicillamine (an anti-rheumatoid drug) downregulates the expression levels of CSE. It is proposed that this inhibition contributes to its efficacy against RA [67]. These findings suggest that H_2_S plays an important role in mediating inflammation in joints. On the other hand, slow H_2_S-releasing compounds show anti-inflammatory activity against joint inflammation. ACS 15 (a slow H_2_S-releasing derivative of the NSAID diclofenac) shows strong anti-inflammatory activity against carrageenan-induced hind-paw edema in comparison to its precursor NSAID [47]. In RA, SPRC also shows anti-inflammatory activity by ameliorating the Nrf2-ARE (nuclear-factor-E2-related factor-2) signaling pathway [31]. In joint inflammation, H_2_S regulates inflammation through several mechanisms, including (1) decreasing the expression of pro-inflammatory mediators, e.g., interleukins (IL) and tumour necrosis factor (TNF), etc.; and (2) via the inhibition of important signaling pathways, including phosphatidylinositol 3-kinase (PI3K), mitogen-activated protein kinase (MAPK), and nuclear factor-κB (NF-κB). The anti-inflammatory action of H_2_S in OA patients depends on both the timing of H_2_S administration and its concentration. Furthermore, whether the action of H_2_S in RA is pro- or anti-inflammatory is debatable [68,69]. There is mounting evidence suggesting a correlation between H_2_S production and the development of bone. The anti-inflammatory action of H_2_S in OA has been observed. It has been found that the levels of H_2_S, 3-MST mRNA expression, and proteins were decreased in OA patients compared to healthy individuals. The levels of other H_2_S-producing enzymes (CSE and CBS) were, however, not affected. These findings suggest that a significant decrease in the production of 3-MST in the cartilage of OA patients may contribute to the decreased production of H_2_S. This, in turn, may lead to the development of OA [70]. Another H_2_S donor GYY4137 (a modified form of Lawesson’s reagent) gained attention in scientific research due to its slow H_2_S-releasing ability and sustainability in aqueous solution in comparison to sulfide salts [71]. In in vitro studies, GYY4137 has been reported to have strong anti-inflammatory and cartilage-protective effects in human synoviocytes (HFLS) and articular chondrocytes (HAC). In complete Freund’s adjuvant (CFA)-induced joint inflammation in mice*,* the anti-inflammatory effects of GYY4137 are associated with the timing of administration [72]. In rats, the levels of H_2_S were increased 30 min after the administration of GYY4137 and persisted for approximately 3 h [73]. Allicin (a sulfur-containing compound in garlic) exhibits anti-inflammatory properties and can act as a therapeutic agent for joint inflammation [74]. ATB-346 (naproxen-derived H_2_S donor) is also useful to alleviate pain induced by OA [75]. Slow H_2_S-releasing compounds such as AP39 have been developed, which particularly affect mitochondrial function. During oxidative stress, AP39 has been reported to show antioxidative and cytoprotective effects. Interestingly, these effects of AP39 are observed at a concentration between 30 to 100 nM, which is considerably lower in comparison to GYY4137, NaHS, and Na_2_S. This shows that the efficacy of AP39 is higher than other H_2_S donors even at lower concentrations [76]. This finding suggests the therapeutic potential of H_2_S-releasing compounds that particularly affect mitochondrial function. Therefore, development of H_2_S-based drugs can contribute to the treatment of joint inflammation.

### 5.2. H_2_S and Sepsis

Sepsis is defined as a serious life-threatening complication that results in organ dysfunction due to dysregulation of the host’s immune response to an infection [77]. It has been reported that development of sepsis is not only confined to the recognition of microbial or host patterns and inflammation. It also severely affects microcirculation, immune tissues, endothelial tissues, microglial cells, neurons, and parenchymal tissues [78,79]. Sepsis is considered to be one of the leading causes of death, which usually affects about 30 million people annually. Sepsis remains a major health concern globally due to the increasing incidence in recent years [80,81]. Sepsis is a systemic disorder that affects the vascular system, cardiovascular system, lungs, central nervous system, and liver [82]. The role of H_2_S in sepsis has been widely studied, and it has been shown that it plays a significant role in the pathogenesis of this condition. The role of H_2_S in sepsis is, however, subject to a lot of discussion in the field. Some studies found that an increased level of H_2_S in sepsis can exacerbate inflammation and organ damage, whereas controlled H_2_S release can show protective effects. It was observed that there is a correlation between sepsis and H_2_S levels. In rodents, cecal ligation and puncture (CLP) is a standard and clinically relevant model to induce sepsis. Following CLP-induced sepsis in mice, increased levels of plasma H_2_S and upregulation of the expression of CSE were observed. It was found that administration of PAG (prophylactically and therapeutically) in CLP-induced mice reduced MPO activity and changes in lung and liver tissues and improved sepsis-associated inflammation. On the other hand, following treatment with NaHS, sepsis-induced organ damage was exacerbated [83]. Administration of dexamethasone in a mouse model of LPS (lipopolysaccharide)-induced endotoxemia significantly decreased the activity of iNOS, the expression of CSE, and ultimately the plasma levels of H_2_S [84]. In mice with CLP-induced sepsis, increased levels of H_2_S-activated NF-κB signaling pathways were observed. This increase in turn upregulated the expression of chemokines (MCP-1 and MCP-2), adhesion molecules (P-selectin, E-selectin, ICAM-1, and VCAM-1), and cytokines (TNF-α, IL-1β, and IL-6) [85]. Furthermore, it was shown that the ERK pathway played a significant role in activating the NF-κB signaling pathway during sepsis. Treatment with ERK inhibitor (PD98059) inhibited the activity of NF-κB and sepsis-induced systemic inflammation, as well as decreasing organ damage. Inhibition of CSE with PAG administration reduced the levels of H_2_S and showed a better prognosis in the liver and lungs, whereas higher H_2_S levels caused a hemodynamic collapse [86]. In an in vitro study, an effect of treatment with FW1256 (slow-H_2_S releasing compound) to the LPS-treated mouse macrophage cell line RAW 264.7 was observed. It was found that FW1256 decreased the production of IL-6, COX2, PGE_2_, iNOS, TNF-α, and IL-Iβ and also inhibited the activity of NF-κB [87]. Moreover, in LPS-treated THP-1 macrophages, prophylactic treatment with different doses of NaHS (0.01 mM, 0.1 mM, and 1 mM) has been reported to decrease pro-inflammatory cytokine production. The anti-inflammatory effect of NaHS was attributed to epigenetic modifications in histones [88].

Administration of NaHS to the cell line (U937) showed upregulation of pro-inflammatory cytokines, including IL-1b, IL-6, and TNF-a, through ERK and NF-кB signaling pathways [48]. Furthermore, in another study, treatment of cell line RAW264.7 with LPS showed overexpression of CSE, chemokines, and cytokines, whereas CSE gene silencing decreased the production of these pro-inflammatory mediators [89]. Pro-inflammatory roles of H_2_S were also observed in LPS-induced endotoxemia in rats and mice [90,91]. In LPS-induced endotoxemic rats, ACS-15 showed anti-inflammatory actions by preventing the production of endogenous H_2_S [92]. This finding suggests that slow H_2_S-releasing donors can exhibit anti-inflammatory actions by blocking the synthesis of endogenous H_2_S through negative feedback [93]. GYY4137 also shows anti-inflammatory actions against LPS-induced endotoxemia in the rat model by inhibiting NF-кB activation, iNOS, and COX_2_ expression. Treatment with Na_2_S_2_O_3_ also decreased liver and lung injury and neuroinflammation and preserved mitochondrial functions in the mice model. The anti-inflammatory effects of Na_2_S_2_O_3_ were associated with the presence of CSE, as in CSE^−/−^ mice, Na_2_S_2_O_3_ did not exhibit anti-inflammatory effects. This finding points to the role of CSE in regulating the protective actions of Na_2_S_2_O_3_ [94]. Furthermore, we have shown that liver sinusoidal endothelial cells (LSECs) also contribute to the pro-inflammatory effects of H_2_S in sepsis. The liver sinusoid is important for maintaining the function of the liver, but its homeostasis is disturbed in endotoxemia and sepsis. It is believed that in endotoxemia H_2_S shows vasoconstriction in liver sinusoids [95,96]. In wild-type (WT) mice, increased gap formation and defenestration in LSECs was observed due to increased H_2_S levels compared to CSE knockout mice. It was also observed that a key factor in H_2_S-induced liver sinusoidal dysfunction is the activation of the substance P-tachykinin receptor 1 axis and the ERK1/2-NF-κB p65 pathway [97,98]. Furthermore, in septic patients and in mice with LPS-induced endotoxemia, similar dysregulation of H_2_S has been observed [99,100,101].

Substance P (SP) and neurogenic inflammation are important mediators that contribute to the inflammatory action of H_2_S in sepsis. Treatment with capsaicin (responsible for the depletion of SP from sensory neurons) alleviates H_2_S-induced lung inflammation in mice. In sepsis, the levels of SP and the expression of pulmonary PPT-A (preprotachykinin A—the gene encoding SP) are upregulated. Administration of PAG downregulates the expression of the PPT-A gene and the levels of SP in lungs, but NaHS administration elevates SP levels [102]. Furthermore, PPT-A gene deletion and prophylactic treatment with SP inhibitor (L703606) shows that SP interacts with H_2_S and plays an important role in the pro-inflammatory effects of H_2_S. Moreover, H_2_S triggers systemic inflammation and multiple organ dysfunction in sepsis through transient TRPV-1 (transient receptor potential vanilloid-1)-mediated neurogenic inflammation [103]. We have shown that in sepsis, H_2_S regulates TRPV-1-mediated neurogenic inflammation by increasing the production of SP and by activating the ERK–NF-κB signaling pathway [104]. In sepsis, H_2_S activates TRPV-1, which in turn upregulates the PGE metabolite and COX_2_ levels, resulting in acute lung injury [105]. However, treatment with capsazepine, an antagonist of TRPV-1, also mitigates lung inflammation induced by H_2_S. These findings suggest that SP and neurogenic inflammatory pathways play an important role in inflammation caused by H_2_S [106].

These studies highlight the complex and biphasic role of H_2_S in sepsis, where it can show either pro- or anti-inflammatory effects. These effects depend upon several factors, including H_2_S release rate, concentration, and its interaction with specific signaling pathways and the cellular environment.

**Figure 3:**
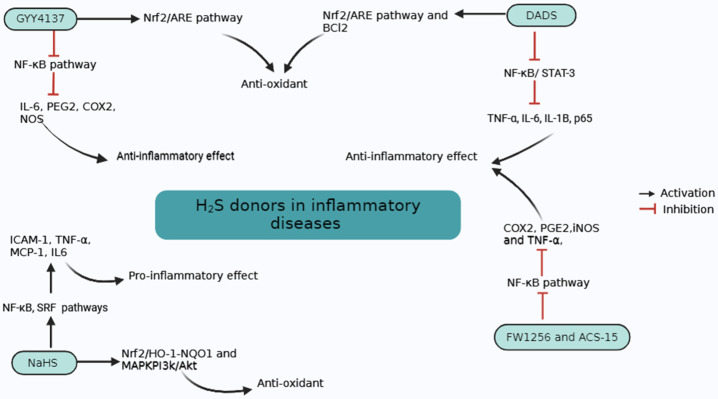
Mechanisms of action of H_2_S donors in inflammatory diseases. GYY4137 and DADS inhibit NF-κB pathways and activate the Nrf2/ARE pathway, which inhibits pro-inflammatory cytokines and activates antioxidant pathways, respectively. NaHS activates NF-κB and SRF pathways and activates pro-inflammatory mediators. NaHS also activates antioxidant pathways. FW1256 and ACS-15 also suppress the NF-κB pathway and show anti-inflammatory effects.

Figure 3 summarizes our current understanding of the role of H_2_S in inflammatory diseases and mechanisms of action of H_2_S donors in these diseases.

### 5.3. Acute Pancreatitis

Excessive consumption of alcohol, gallstones, and increased levels of triglycerides in the blood usually result in acute inflammation of the pancreas, known as acute pancreatitis (AP) [107]. This condition is generally associated with increased circulating levels of digestive enzymes (amylase and lipase) and abdominal pain [108,109]. Inflammatory responses in severe cases of AP trigger both localized as well as systemic complications, which makes the treatment of AP even more complicated [110,111]. Localized pathological hallmarks of AP include infected necrosis, acute necrotic collections (ANCs), acute peripancreatic fluid collections (APFCs), pseudocysts, and walled-off necrosis (WON). Systemic pathological hallmarks of severe AP are shock, and kidney and respiratory failure. In AP, the most common contributing factor to death is believed to be the multiple organ dysfunction syndrome (MODS) [112]. A major component of MODS is lung injury, that is clinically manifested as acute respiratory distress syndrome (ARDS). It has been observed that production of H_2_S is significantly increased in AP. H_2_S acts as a pro-inflammatory mediator in AP due to the abundance of CSE and CBS in the pancreas [113]. Different approaches (such as silencing of the CSE gene by siRNA, inhibition of H_2_S synthesis by pharmacological agents, and deletion of the CSE gene) were used to check the pro-inflammatory effects of H_2_S in AP [114]. In an in vivo study in cerulein-induced AP (cerulein is a cholecystokinin analogue commonly used to induced pancreatitis) in mice, inhibition of CSE by PAG decreased H_2_S synthesis, which led to decreased inflammation. This decrease in inflammation was associated with the downregulation of MIP-1α, MIP-2, and MCP-1 [22]. Furthermore, it was observed that H_2_S may exacerbate AP by the PI3K/Akt/Sp1 signaling pathway [115]. In addition to H_2_S, levels of ammonia in plasma and tissues were also elevated in severe cases of AP. CBS also plays a role in the progression of AP. In in vivo experiments, AOAA administration to mice helps to mitigate AP and associated acute lung injury [116]. Treatment with slow H_2_S-releasing compounds, including SPRC, and S-diclofenac, showed anti-inflammatory effects against pancreatic and lung injury during AP. The protective role of these compounds can be due to the suppression of the NF-κB signaling pathway [32,46]. Administration of NaHS leads to the activation of ICAM-1 expression and the adhesion of neutrophils to the isolated acinar cells treated with cerulein. This process is mediated by the activation of NF-κB and Src-family kinase signaling pathways [49]. In addition to cytokines, adhesion molecules, and chemokines, H_2_S also plays a key role in inflammation associated with AP through its interaction with SP. Treatment with NaHS showed a significant increase in levels of SP in the plasma and inflammation in lungs in a mouse model of AP [106]. In PPT-A^−/−^ mice, however, there was significant protection against AP-associated lung inflammation. In addition, administration of CP-96345, an antagonist of neurokinin-1 receptor-NK-IR (SP receptor) showed a protective response against lung inflammation triggered by H_2_S. Administration of PAG decreased the elevated levels of SP in the lungs, pancreas, and plasma in severe cases of AP. In addition, in mice with AP, PAG treatment also decreased the levels of expression of NK-1R and PPT-A in the lungs and pancreas. These findings indicate that the pro-inflammatory effects of H_2_S in AP can be regulated through SP [117]. Additionally, studies with isolated pancreatic acini suggest that H_2_S enhances the Toll-like receptor 4 pathway in AP and NF-κB activity through SP [118]. These studies show that H_2_S plays a crucial role in the progression of AP, and it can act as a potential therapeutic target. 

## 6. H_2_S and Diseases of the Cardiovascular System

CVD (cardiovascular disease) includes all diseases related to the heart and the vascular system. CVDs are considered to be the leading cause of death globally, which results in an increased economic burden [119]. CVD can be caused by physical inactivity, a sedentary lifestyle, and poor dietary patterns, etc. [120]. A substantial body of evidence has revealed that H_2_S can improve and play a protective role in many diseases, including atherosclerosis, cardiac hypertrophy, ischemia–reperfusion injury, myocardial infarction, cardiac arrhythmia, and heart failure (HF) [121]. It has been reported that H_2_S has a beneficial role in cardiovascular diseases. H_2_S plays a cardioprotective role by regulating several mechanisms, such as alteration in mitochondrial autophagy, inhibiting pro-inflammatory cytokines, inhibiting endothelial mesenchymal transition, and preventing lipid peroxidation and antioxidative stress. [122,123]. H_2_S is a key regulator in maintaining the oxidation/reduction balance by neutralizing ROS in CVD. It shows antioxidant properties by altering L-cysteine amino acid residues at different signaling molecules, including HIF-1α, keap1/Nrf2, and NF-κB [124]. Ischemia–reperfusion (I/R) injury often leads to the destruction of myocardial tissues and eventually HF. Reperfusion helps in the treatment of ischemia but also activates a complex series of reactions that cause oxidative damage and inflammation, which eventually causes cell injury [125]. It has been reported that the exogenous administration of H_2_S in ischemic rat hearts resulted in the activation of the JAK2 pathway, which increased the phosphorylation and activation of STAT3 [126]. In in vivo experiments, prophylactic treatment with sulfur dioxide (SO_2_) decreased I/R-induced heart damage, while elevated antioxidant activity and upregulation of CSE expression were observed [127]. In a clinical study, individuals with ACS (acute coronary syndrome) showed significantly lower levels of H_2_S. Expression of CX3CL1 and CCL2 (chemokines) was upregulated as compared to non-CAD (coronary artery disease) and angina patients [128]. Furthermore, an increased risk of CVD was observed in chronic hemodialysis patients due to the activation of protein kinase C beta II (PKCβII), overexpression of adhesion molecules (VCAM-1 and ICAM-1), and downregulation of CSE [129]. In ApoE knockout mice and Ox-LDL-stimulated human aortic endothelial cells (HAEC), aortic and plasma H_2_S levels were reduced during atherosclerosis [130]. An effect of H_2_S donor (NaHS) and endogenous H_2_S inhibitor (PAG) in atherosclerosis has been reported. In an in vivo study, treatment with NaSH significantly increased the levels of plasma H_2_S in the ApoE^−/−^ mouse model. This increase in plasma H_2_S levels reduced the size of atherosclerotic plaques and levels of ICAM-1 in plasma and the aorta, which improved atherosclerosis. On the other hand, treatment with PAG decreased the levels of plasma H_2_S. This decrease in plasma H_2_S levels increased plaque size and levels of ICAM-1 in plasma and exacerbated atherosclerosis. In an in vitro study of TNF-α treated human umbilical vein endothelial cells (HUVECs), prophylactic treatment with NaHS inhibited the expression of ICAM-1 [52]. In rats, the release of myocardial enzyme and myocardial infarct size was prevented by H_2_S. This prevention was due to the inhibition of oxidation, activation of Sirt1/PGC1α signaling pathways, and attenuation of the activation of inflammatory cytokines [131,132]. Furthermore, in in vivo experiments, H_2_S showed a protective role in the development of myocardial injury by angiogenesis through activating the vascular endothelial growth factor (VEGF) and hindering the activation of parastatin [133]. It was found that plasma and aortic H_2_S levels were significantly decreased in the high-fat diet (HFD) cardiomyopathy mouse model [134]. Cardiac remodeling is influenced substantially by elevated levels of homocysteine in blood (hyperhomocysteinemia). Hyperhomocysteinemia plays a role in the progression of myocardial hypertrophy by the formation of the MEF2C-HDAC1 (myocyte-specific enhancer factor 2C/histone deacetylases) complex. This complex leads to the inactivation of MEF2C and suppresses miR-133a expression in the heart muscles. On the other hand, H_2_S activates MEF2C and initiates miR-133a, which results in the prevention of cardiac hypertrophy [135]. In in vivo experiments, it was observed that mice deficient in CBS (CBS^+/^^−^) showed upregulation of CSE expression, which suggests that a negative correlation exists between the regulation of these two enzymes [136]. Prolonged activation of angiotensin II (a hormone responsible for regulating blood pressure) can initiate cardiac remodeling. Renin-angiotensin-aldosterone-based pharmacological agents are used to treat cardiac remodeling. Among these agents, angiotensin receptor blockers and ACE (angiotensin-converting enzyme) inhibitors are commonly used [137]. H_2_S inhibits the activity of angiotensin II in heart tissues, which mitigates myocardial hypertrophy and fibrosis [138]. It has been reported that Cx43 levels in cardiomyocytes were upregulated by endoge-nous H_2_S, suggesting that H_2_S contributes to the maintenance of arrhythmia and car-diac health [139]. Angiotensin II activates the Kruppel-like factor 5 (KLF5) gene, which in turn results in cardiac remodeling. KLF5 is a key regulator in cardiac remodeling and affects different conditions, such as angiogenesis, cardiac fibrosis, thickening of arterial walls, and enlargement of the heart. In an in vivo study, a decrease in the thickening of the arterial wall was observed in vascular-induced injury in KLF5^−/−^ mice. In addition, H_2_S downregulates the expression of the KLF5 gene, which results in alleviating cardiac hypertrophy [140]. In in vitro studies, upon the exposure of human atrial fibroblasts (HAF) to H_2_S, a decrease in arterial fibrosis was observed [141]. At a molecular level, the CSE/H_2_S pathway modulates eNOS expression by the PKC βII/Akt signaling pathway and shows cardioprotective effects against atherosclerosis [142]. CBS gene mutations also play an important role in the progression of CVD and endothelial dysfunction by interacting with the mitochondrial function [143]. A transmembrane protein Cx43 (connexin) is primarily present in the ventricle and exhibits a significant link with cardiac arrhythmias [144]. Moreover, plasma and myocardial levels of H_2_S were decreased during cardiac failure. Furthermore, after transverse aortic constriction (TAC), increased abnormal cardiac function and dilation were seen in CSE^−/^^−^ mice in comparison to CSE^+/^^+^ mice. On the other hand, after TAC, CSE overexpressing-mice exhibited cardioprotective effects by using endogenous H_2_S. Due to the limited research, however, there is no evidence concerning the expression of 3-MST and CBS transgenic mice [10]. In in vivo experiments, upon exposure to 4-carboxyphenyl isothiocyanate (4-CPI), a significant decrease in ventricular arrythmias and myocardial infarct size was observed in rat heart [145]. In addition, treatment of isolated rat hearts with another H_2_S donor AP39 attenuated IR injury by reducing harmful oxidative molecules and blocking the opening of mitochondrial permeability transition pore (PTP) [146]. Alpha lipoic acid (ALA), a novel organosulfur compound, also showed cardioprotective effects in post-I/R arrhythmias by regulating K_ATP_ channels. This effect of ALA can be due to the direct release of sulfur and H_2_S together [147]. In isolated pig and mice hearts, zofenopril (ACE inhibitor) alleviates myocardial infarct size (post-IR injury) by increasing H_2_S and NO production [148]. Administration of NaHS in rabbit hearts activated the cGMP/PKG signaling pathway, which helped in decreasing the size of myocardial infarcts [50]. In mice with left coronary artery (LCA) occlusion-induced HF, improved left ventricular function and a significant decrease in myocardial hypertrophy were seen after administration of Na_2_S [149]. Administration of SPRC upregulated the expression of CSE and increased the H_2_S concentration, which showed protective effects against myocardial infarction by decreasing ROS production [150]. Furthermore, another H_2_S donor GYY4137 has been reported to provide cardioprotective effects in atherosclerosis by regulating the PI3K/Akt/TLR4 signaling pathway [34]. GYY4137 shows beneficial effects by inhibiting myocardial fibrosis, reducing oxidative stress and inflammation [151]. In high fructose-fed insulin-resistant rats, it was demonstrated that administering a freshly prepared garlic homogenate, known to produce H_2_S upon interacting with cellular proteins, activates the myocardial Nrf2 via the PI3K/Akt pathway. This activation helps reduce cardiac hypertrophy and oxidative stress by enhancing the antioxidant defense system [41]. A novel H_2_S donor SG1002 maintained vascular homeostasis by preventing mitochondrial dysfunction and improving myocardial vascular density in individuals with HF [152]. Recently, it has been found that SG1002 upregulates the expression of H_2_S-producing enzymes, which activates the nuclear Nrf2 pathway. Activation of the Nrf2 pathway decreases the oxidative stress as well as ROS formation and protects against cardiac hypertrophy and HF [153]. Furthermore, in ACS patients, intravenous administration of Na_2_S_2_O_3_ (inorganic sodium salt with thiosulfate ions–a clinically approved drug) up to 15g is considered safe [154]. Many novel therapeutic targets, such as mitofusin 2 (Mfn2-mitochondrial protein), still need to be explored in CVD. H_2_S plays a significant role in regulating Mfn2, and its abnormal function can result in IR, dilated cardiomyopathy, and HF [155,156]. Although H_2_S is known to regulate Mfn2, currently there are no data on the use of H_2_S donors specifically in the treatment of Mfn2-related cardiovascular diseases. Targeting this protein with H_2_S donors could be an important and innovative area for future research [157].

**Figure 4:**
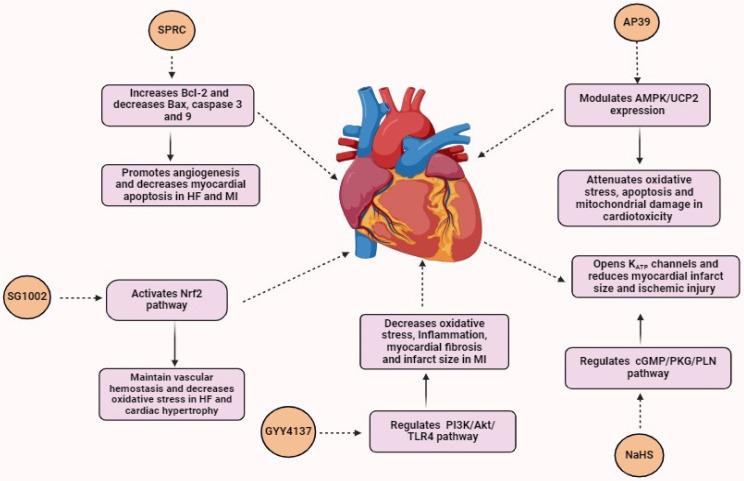
Role of H_2_S donors in CVD. SPRC promotes angiogenesis and decreases myocardial apoptosis in HF and MI. SG1002 maintains vascular hemostasis and decreases oxidative stress in HF and cardiac hypertrophy. GYY4137 decreases oxidative stress, inflammation, myocardial fibrosis, and infarct size in MI. NaHS opens up K_ATP_ channels and reduces myocardial infarct size and ischemic injury. AP39 attenuates oxidative stress, apoptosis, and mitochondrial damage in cardiotoxicity.

Figure 4 summarizes our understanding of the role of H_2_S in CVD and the mechanism by which it acts.

## 7. H_2_S and Viral Diseases

Viral infections are considered a major cause of mortality as well as a major economic burden globally in the clinical setting. Members of the Paramyxoviridae family, such as hMPV (human metapneumovirus), RSV (respiratory syncytial virus), and NiV (Nipah virus, a zoonotic virus) contribute to major respiratory tract infections. These infections include pneumonia, asthma progression, bronchiolitis, and cough [158,159]. Despite the severe infections caused by these contagious viruses, currently there are no vaccines or effective therapeutic strategies except prophylactic immunization for RSV. It was found that in AECs (airway epithelial cells) these viruses activate several regulators of inflammation, such as cytokines and chemokines, which contribute to disease development [160]. In infected cells, gene expression of these regulators is mediated by the activation of IRF-3 (interferon regulatory factor 3) and NF-κB signaling pathways. In in vitro experiments, AECs were infected with RSV to check the role of endogenous H_2_S levels in viral infections. In RSV-infected AECs, the expression of CSE was downregulated while the decomposition of H_2_S was increased compared to normal cells. Furthermore, increased expression of pro-inflammatory cytokines and viral particles was measured after the inhibition of endogenous H_2_S by PAG. This finding sheds light on the protective role of endogenous H_2_S in viral replication. The underlying mechanism of endogenous H_2_S in preventing viral infection is not clearly understood. It has been observed that in viral infections, levels of Nrf2–ARE pathway-associated antioxidant enzymes and ROS are significantly increased, which results in impaired antioxidant responses. To control the infections caused by respiratory viruses, activation of the Nrf2 pathway and induction of long-term antioxidant activity can be a potential strategy [161]. H_2_S activates several antioxidant mechanisms, including NADPH oxidase enzyme inhibition and increased activation of Nrf2 translocation to the nucleus. It suggests the role of endogenous H_2_S in protecting against pulmonary viral infections. Despite directly activating antioxidant mechanisms, H_2_S also contributes to antioxidant activity through various indirect effects. For example, increased levels of glutathione (GSH) (an important compound with antioxidant activity as well as an efficient scavenger of ROS) are maintained by endogenous H_2_S [162]. GSH is reported as an important compound to inhibit viral infections. However, in HSV-1 (herpes simplex virus type 1), levels of GSH were surprisingly decreased, whereas after GSH administration, viral replication was inhibited [163]. Recently, it has been highlighted that GSH shows therapeutic potential against COVID-19 by interacting with human proteins such as TMPRSS2 (transmembrane serine protease 2) and ACE2 (angiotensin-converting enzyme 2). These proteins are important in the adhesion and entry of viral cells in a host cell [164]. Furthermore, a close relationship between endogenous H_2_S levels and mortality rate among 74 SARS-CoV-2 patients has been observed. The findings of this study suggest that levels of H_2_S were significantly elevated on days 1 and 7 in patient survivors on the 28th day compared to non-survivors. Specifically, a total sulfide pool (which measures H_2_S levels) below 150.44 μM at the outset was associated with an elevenfold increase in the risk of dying within 28 days. A reduction in H_2_S levels of more than 36% from day 1 to day 7 correlated with higher rate of 28-day mortality. This finding indicates that lower H_2_S levels during SARS-CoV-2 infection may exacerbate the infection [165]. In RSV-infected mice, the administration of GYY4137 reduced the activation of different factors, including GM-CSF (granulocyte-macrophage colony-stimulating factor) and G-CSF (granulocyte-colony stimulating factor). However, a decrease in levels of pro-inflammatory mediators, including TNF-α, IL-6, IL-1, KC, IFN-α, IFN-β, MCP-1, MIP-1α, and -β was also observed. In in vivo experiments, in RSV-infected mice a significant decrease in RSV titer and neutrophilia in lungs was observed after the treatment with GYY4137 in comparison to normal controls [166]. Treatment with DADS has elevated the level of GSH in lung tissues and prevented the release of pro-inflammatory cytokines (IL-6, IL-8, and TNF). Inhibition of pro-inflammatory cytokines was linked to decreased recruitment of inflammatory cells in the lungs notably reducing neutrophil infiltration [44]. In mice with LPS-induced acute lung injury, it has been observed that sulforaphane (novel H_2_S donor belonging to isothiocyanate) inhibited the release of pro-inflammatory cytokines [167]. The transcription factor Nrf2 played an important role in the lung protection mediated by sulforaphane by enhancing mitochondrial function and energy metabolism. These findings suggest that exogenous administration of H_2_S can protect host cells from severe viral infections [168]. Although various studies have indicated the antiviral effects of H_2_S, these effects are not universal. For example, rotavirus infection was not inhibited by XM-01 (a cysteine-based perthiol derivative), which suggests that H_2_S does not have a protective effect against non-enveloped viruses [169]. This limitation is associated with the mechanism of action of XM-01, i.e., by inhibition of membrane fusion (enveloped viruses), whereas non-enveloped viruses penetrate host cells via endocytosis. These results suggest that H_2_S can be considered a potential therapeutic target against infections caused by enveloped viruses. The role of H_2_S has also been studied in several other respiratory tract infections [170]. In rats, administration of NaHS significantly improved ventilator-induced lung injury (VILI) by inhibiting ATF4 nuclear expression and PERK phosphorylation. In L2 cells, NaHS treatment decreased the endoplasmic reticulum (ER) stress and autophagy, which improved VILI [171]. During cellular stress conditions, 4-PBA (4-phenylbutyric acid; ER-stress inhibitor) and NaHS inhibited the activation of ERK, NF-κB, p65, MAPK, p38, and JNK pathways [172]. H_2_S alleviates VILI by neutralizing the generation of ROS through the PI3K/Akt signaling pathway [173]. H_2_S also plays a significant role in pulmonary physiology and in the development of asthma. Decreased enzymatic H_2_S production can exacerbate the onset of asthma [174]. On the other hand, high doses of H_2_S (300 ppm) can enter blood circulation through the pleural membrane and result in vascular changes and hypoxemia [175]. In clinical studies, it has been found that exogenous administration of H_2_S downregulates the phosphorylation of p38 and ERK1/2 pathways. Inhibition of these pathways results in the suppression of pro-inflammatory mediators and cell proliferation [176]. Furthermore, clinical trials have revealed that higher levels of serum H_2_S are linked to improved lung function (as measured by forced expiratory volume 1.0) in asthma patients. Higher levels of H_2_S in serum were associated with lower numbers of neutrophils in the sputum, which indicates reduced inflammation in the airways of asthma patients [177]. However, Na_2_S_2_O_3_ also shows antiviral and anti-inflammatory activities through activating the Nrf2/ARE pathway. This compound is widely used in the treatment of calciphylaxis and cyanide poisoning. It can also serve as an important therapeutic agent for the treatment of SARS-CoV-2 [178]. In children, Na_2_S_2_O_3_ significantly reduced acute lung injury associated with pneumonia [179]. Furthermore, Na_2_S_2_O_3_ inhalation also decreased acute lung injury associated with SARS-CoV-2 infection [180]. These findings suggest that the development of H_2_S-based therapeutics can be a potential target in the treatment of viral infections. 

**Figure 5:**
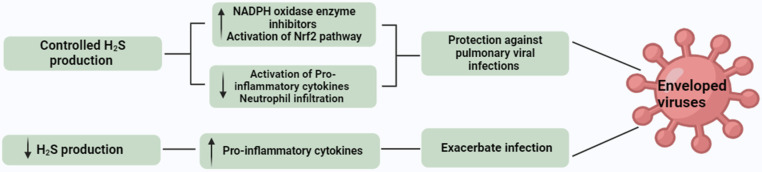
Role of H_2_S in viral infections. Controlled production of H_2_S provides protection against pulmonary viral infections. Decreased H_2_S production exacerbates infection.

Figure 5 summarizes our current understanding of the role of H_2_S in viral infections.

## 8. H_2_S and CNS Diseases

Central nervous system-related diseases are usually caused by any damage to peripheral nerves, the brain, spinal cord, and neurodegeneration. Globally, these diseases are considered to be the second major cause of morbidity as well as mortality and affect billions of people annually [181]. Neurodegenerative disorders such as Huntington’s disease (HD), Alzheimer’s disease (AD), and Parkinson’s disease (PD) are characterized by the degeneration of nerve cells. CBS and 3-MST are key enzymes for the production of H_2_S in CNS, while 90% of H_2_S is synthesized by 3-MST in the brain [182]. 3-MST is abundantly present in neurons, and it is responsible for the regulation of mitochondrial H_2_S for neurodegenerative processes [183]. CBS, on the other hand, is present in supporting cells, i.e., microglia, astrocytes, and oligodendrocytes. CBS is responsible for neurological functions, including synaptogenesis, maintaining the permeability of the blood–brain barrier, and neurogenesis [184,185]. H_2_S increases long-term potential (LTP) by activating N-methyl-D-aspartate (NMDA) receptors in neurons. NMDA receptors help in preventing oxidative damage and dysfunction of mitochondria through ROS and neutralizing free radicals [186]. H_2_S also triggers calcium homeostasis in astrocytes, regulates apoptotic signaling in neurons, and activates anti-inflammatory responses in microglia and astrocytes [187,188]. Findings of these studies emphasize that H_2_S acts as a neuromodulator and shows a protective response against neurodegenerative disorders. These neuroprotective effects are due to its anti-neuroinflammatory, antioxidant, and anti-apoptotic properties. AD is a neurological disorder that is associated with impaired cognitive functions and loss of memory. This condition commonly occurs due to pathological changes such as increased phosphorylation of tau proteins and accumulation of β-amyloid plaque in the brain and correlated with oxidative stress [189]. It has been suggested that amyloid-beta (Aβ) inhibits EAAT3-excitatory amino acid transporter 3 (transporter of cysteine), which disrupts H_2_S signaling. Consequently, this disruption in H_2_S signaling leads to neuronal degeneration and oxidative stress [190,191]. It has been reported that in AD individuals, levels of H_2_S in the brain and plasma were significantly reduced in comparison to the normal control group, which emphasizes the neuroprotective role of H_2_S in AD [192]. However, a negative correlation exists between levels of plasma H_2_S and the progression of AD. In the trans-sulfuration pathway, H_2_S is produced from homocysteine by the catalytic action of CBS, whereas SAM acts as a co-factor for CBS [193]. Although in AD patients, levels of homocysteine were higher, and levels of SAM were lower, no difference in the levels of CBS between AD patients and normal individuals was observed. These findings indicate that lower levels of H_2_S may be due to the reduced activity of CBS, which supports the hypothesis that the CBS-H_2_S signaling pathway is affected by AD [194,195]. In a mouse model of AD, it was found that H_2_S shows neuroinflammatory effects through different pathways, for example, by inhibiting phosphorylation of STAT3, preventing NF-κB activation pathways in the hippocampus, triggering cathepsin S (Cat S), and maintaining the function of mitochondria by the JNK-MAPK signaling pathway in microglia [196,197]. Contributions of CSE and CBS to health and disease are relatively well documented, but more research is needed to explore the role of 3-MST in pathophysiological conditions. In the AD mouse model, it has been observed that the 3MST/H_2_S pathway plays a significant role in memory and cognitive function. In the hippocampus and cortex regions, the activity of 3-MST was significantly decreased. Treatment with 3-MST substrate (sulfanegen) significantly protected against memory loss and cognitive impairment caused by 3-MST [198]. Another neurodegenerative disorder caused by a deficiency in dopaminergic neurons in the SN (substantia nigra) and loss of dopamine in the striatum is termed PD [199]. Cognition impairment is commonly caused by neuro-pathological hallmark events, such as the deposition of misfolded proteins, oxidative stress, abnormal mitochondrial function, and neuroinflammation [200]. A substantial body of evidence suggest that H_2_S protects PD patients from oxidative stress by exerting an antioxidant effect [201]. In a mouse model of PD, it was found that H_2_S regulates mitochondrial function by activating the JNK-MAPK pathway, which alleviates cell apoptosis [202]. Controlled production of CBS-mediated H_2_S exerts anti-neuroinflammatory effects in astrocytes, Whereas increased synthesis of H_2_S produced from gut microbiota releases cytochrome C, which elevates α-synuclein fibril production and triggers the accumulation of ROS [203,204]. These findings suggest that the neuroprotective effect of H_2_S is concentration-dependent. Another neurodegenerative disorder characterized by a mutation in the huntingtin gene and its deposits in neurons, which specifically targets corpus striatum, is known as HD [205]. Destruction in the corpus striatum affects the motor activity of the brain and results in cognitive impairment, involuntary movements of muscles (such as chorea), and changes in behavior [206]. Defective huntingtin proteins affect many cellular as well as metabolic activities, such as mitochondrial activity, DNA repair, redox homeostasis, nuclear-cytoplasmic transport, autophagy, transcription, and translation [207,208,209]. Among these processes, H_2_S regulates several, and many studies have shown that disruption in cysteine metabolism causes HD. Despite many treatment options being available for patients, the prognosis of neurodegenerative diseases is compromised. The neuroprotective role of H_2_S specifically in 3xTg (triple transgenic—PSEN1 mutation M146V, MAPT P301L tau mutation, and Swedish APPKM670/671 NL mutation, a widely used model to induce AD) mice was observed [210]. It was observed that daily intraperitoneal administration of GYY4137 for three months resulted in improvement in both cognitive as well as motor function. Administration of NaHS with sulfur water intraperitoneally for 12 weeks improved brain functions in a 3xTg-AD mouse model [211]. Furthermore, a significant decrease in the deposition of β-amyloid plaques was observed in parts of the brain (hippocampus and cortex). This decrease was associated with the inhibition of ERK, P38, and JNK signaling pathways against hyperphosphorylation of tau proteins and neuroinflammation. Allicin also exhibits neuroprotective effects in AD patients. It inhibits the activity of butyrylcholinesterase and acetylcholinesterase (enzymes responsible for decreasing the levels of acetylcholine and in the progression of AD). In AD, the use of allicin slows down neuronal death, decreases the level of tau proteins, and improves impaired cognitive functions [42,43]. In another study, it was observed that administration of AP39 in APP/PS1 double-transgenic mice (AD model) by intraperitoneal injection for six months daily improved mitochondrial and cellular functions in neurons. Furthermore, AP39 inhibited mitochondrial DNA damage, increased antioxidative activity, and increased the generation of ATP within neurons in comparison to a normal control group [212]. In an in vivo study, in a 6-hydroxydopamine (6-OHDA)-induced PD rat model, it has been found that natural H_2_S levels were reduced in the SN. Conversely, administration of external H_2_S was found to elevate these levels and prevent movement disorders. This was achieved by restoring the levels of tyrosine-hydroxylase-positive neurons in the SN. This effect is potentially due to enhanced leptin signaling and elevated malondialdehyde levels in the striatum. Furthermore, the administration of NaHS protected SH-SY5Y cells from mitochondrial transmembrane potential loss, apoptosis, and oxidative stress induced by the neurotoxin 1-methyl-4-phenylpyridinium (MPP+) in a PD mice model [51]. In the 1980s and 1990s, AOAA was used for the treatment of HD and tinnitus in clinical studies. It was found that AOAA did not show strong therapeutic effects, which limits its development in a clinical setting. However, AOAA did not show any adverse effects during these clinical trials either [213,214]. Abnormal mitochondrial function and oxidative stress are believed to be the main causes of HD, AD, and PD. H_2_S provides protection against neurological disorders through the upregulation of GSH, Nrf2, SIRT-1, GSK3β, and Keap1 and downregulation of pro-inflammatory mediators, JNK-MAPK, and Bax caspase 3/9/12 signaling pathways. Regulation of these pathways provides anti-apoptotic, antioxidative, and anti-inflammatory effects. These pathways can also affect the activities of proteins by sulfhydration [4]. These findings suggest that controlled H_2_S production shows a neuroprotective role, and H_2_S donors can be used as potential agents for treating neurodegenerative disorders.

**Figure 6:**
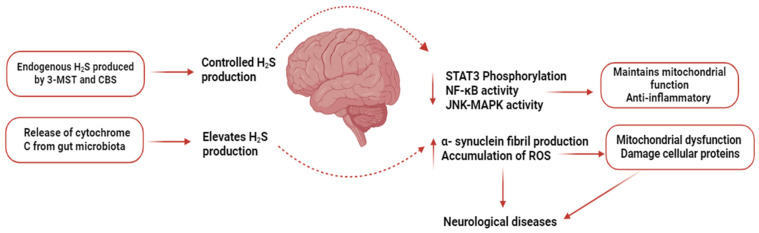
Role of H_2_S in neurological disorders. Controlled H_2_S production helps in the maintenance of mitochondrial function and provides anti-inflammatory effects, whereas increased H_2_S production results in mitochondrial dysfunction and damages cellular proteins.

Figure 6 summarizes our current understanding of the role of H_2_S in neurological disorders.

## 9. Uses of H_2_S Donors in the Clinical Setting

In preclinical studies, H_2_S has been widely recognized for its therapeutic potential in different conditions. However, translating this knowledge to the clinic has been a challenge for researchers due to its pharmacokinetic limitations. For instance, Na_2_S and NaHS are commonly used in research, but their immediate and uncontrollable release limits their usefulness in clinical settings. Organosulfur garlic-containing compounds (DATS and DADS) exert vasoactive properties. Their use in clinical settings is also limited due to their short lifespan and unstable nature [14]. Over the past decades, researchers have developed many H_2_S-releasing compounds for clinical settings that have been tested for their usefulness in different clinical conditions (Table 2). 

## 10. Gaps in Knowledge and the Way Forward

As described above, H_2_S acts as a pro- and anti-inflammatory mediator, antioxidant, and vasodilator. Also, H_2_S significantly contributes to different disease conditions, such as inflammatory conditions, neurological disorders, cardiovascular diseases, and viral infections. The precise mechanisms by which H_2_S significantly affects the host are, however, not clearly understood. Investigating the complex molecular mechanisms of H_2_S in diseases will be helpful for the development of novel H_2_S-based therapeutics. We now have a better understanding of CSE and CBS, but more research is needed to explore the effects of 3-MST as well. We need better H_2_S synthesis inhibitors and donors in order to translate these approaches to clinical settings. Some important questions that need to be addressed as the field develops are related to the following: (1) the concentration of H_2_S to be administered, (2) the timing of administration, (3) the nature of H_2_S-based compounds, (4) the pharmacokinetics of H_2_S-based drugs, and (5) the sensitivity and specificity of H_2_S. 

## 11. Conclusions

The recognition of H_2_S as a critical signaling molecule marks a significant paradigm shift in biomedical research. The diverse roles of H_2_S in health and disease underscore the importance of continued research to fully understand the mechanisms by which it acts and therapeutic potential of this knowledge. The development of H_2_S donors and inhibitors provides promising strategies for treating a wide range of conditions, including inflammatory diseases, cardiovascular diseases, viral infections, and neurological diseases. The translation of H_2_S donors from pre-clinical to clinical settings can lead to novel therapeutic strategies for different diseases. However, further research is needed to assess the dosage of H_2_S donors and inhibitors as well as the timing of intervention. This approach will lead to the translation of knowledge on H_2_S from bench to bedside.

## Figures and Tables

**Table 1:** Mechanism of action and effects of common H_2_S donors in different diseases.

H_2_S Donor	Mechanism of Action	Disease Models	Effects on Cells	References
S-propargyl-cysteine (SPRC)	Ameliorates Nrf2-ARE pathway	In rheumatoid arthritis	Anti-inflammatory	[31]
	Suppression of pro-inflammatory cytokines	In pancreatitis-induced acute lung injury	Anti-inflammatory	[32]
	Decreases the ROS formation, Bax caspase 3 and 9, increase Bcl-2 expression	Myocardial infarction and heart failure	Antioxidant Promotes angiogenesis	[33]
Morpholin-4-ium 4-methoxyphenyl-morp holino-phosphinodithioate (GYY4137)	Inhibition of P13/Akt/TLR4 pathway	Atherosclerosis	Maintain mitochondrial function	[34]
	Suppression of pro-inflammatory cytokines and chemokines	RSV-infected cells	Anti-inflammatory	[35]
	Activation of Nrf2-ARE pathway and suppression of NOS2 and COX-2 expression.	In osteoarthritis	Antioxidant	[36]
	Decreased upregulation of iNOS, COX2, NF-кB, and STAT 3	Endotoxemia	Anti-inflammatory	[37]
	Increased TGF-β expression and decreased IFN-γ and IL-17 production	Multiple sclerosis	Anti-inflammatory	[38]
AP-39	Modulation of AMPK/UCP2 pathway and decrease in ROS	Ischemia–reperfusion injury and cardiotoxicity	Antioxidant Maintain mitochondrial function	[39]
	Decrease in MPO and IL-6 levels, increase in IL-10 levels	Acute lung injury	Anti-inflammatory	[40]
Allicin and Diallyl disulfide (DADS)	Activates Nrf2/ARE pathway	Cardiac hypertrophy and Alzheimer’s disease	Antioxidant	[41,42,43]
	Inhibition of pro-inflammatory cytokines	In viral-associated lung injury	Anti-inflammatory	[44]
	Suppression of p-AKT, NOS2, and PI3K levels	In osteoarthritis	Mitigate pain	[45]
S-diclofenac	Suppression of NF-κB pathway	Pancreatic and acute lung injury in acute pancreatitis	Anti-inflammatory	[46]
	Stabilizes P53, P21, P53AIPI, and Bax	Atherosclerosis	Antiproliferative	[36]
	Inhibition of COX enzymes, NO production, and decreased MPO activity	Carrageenan induced hind-paw edema	Anti-inflammatory	[47]
NaHS	Activation of ERK and NF-κB signaling pathways	Sepsis-induced organ damage	Pro-inflammatory	[48]
	Activation of NF-κB and Src-family kinase signaling pathways	Acute pancreatitis	Pro-inflammatory	[49]
	Activation of cGMP/PKG signaling pathway	Myocardial infarction	Antioxidant	[50]
	Inhibition of mitochondrial transmembrane potential loss	Parkinson’s disease	Relieve mitochondrial dysfunction	[51]
	Inhibits IkB-a degradation and NF-κB nuclear translocation	Atherosclerosis	Decreases expression of adhesion molecules (specifically ICAM-1)	[52]

**Table 2:** Development of H_2_S-based therapeutics in the clinical setting.

Therapeutic Compound	Pharmacological Profile	Targeted Condition	Clinical Trial Phase	References
IK-1001	Injection of Na_2_S (stabilized form)	Mitigation of cardiac complications during coronary artery bypass surgery	Discontinued after phase II	[215]
Na_2_S_2_O_3_	Sodium salt (inorganic) with thiosulfate ions	Cyanide intoxication, cisplatin toxicities, and calciphylaxis in end-stage renal disease	Approved	[179,216]
Zofenopril	ACE inhibitor in conjunction with a H_2_S donor	Hypertension	Approved	[217,218]
GIC-1001	Analgesic effect of trimebutine with H_2_S release	Visceral pain during sedation-free, full colonoscopy	Clinical phase II (pending)	[219]
SG1002	H_2_S-releasing prodrug	Heart failure	Phase I	[152]
ATB-346	H_2_S-releasing naproxen-derived compound	Inflammatory conditions	Phase II	[220]
Alpha lipoic acid	Organosulfur compound	Hypertension, cardioprotection, diabetes, and obesity	Phase II	[221]
ATB-340	H_2_S releasing aspirin-derived compound	Anti-coagulant for CVD	Discontinued	[222]
